# Variola and Vaccinia

**Published:** 1882-04-20

**Authors:** T. B. Greenley

**Affiliations:** Kentucky


### VARIOLA AND VACCINIA.

BY T. B. GREENLEY, M. D., OF KENTUCKY.

As there has, recently, been a great deal said, pro and con, about vaccination as a small-pox prophylactic, I thought I would offer a few words as to my own observations in regard to the matter, as they have been decidedly in favor of vaccination.

Twenty-five years ago I was called to see a negro boy, four years old, who was afflicted with small-pox. His father, a deck-hand on a steamboat, took varioloid and was sent to the pest-house in Louisville for treatment and isolation. Before entirely well, he made his escape from the institution, and visited his wife in Bartlett county, this State, who was owned by a Mr. Samuels. She was the mother of the boy above alluded to. The father, on leaving the hospital, took his old over-coat with him to his wife's house, and she made a pillow of it for the boy to sleep on, at the foot of the bed. In two weeks from that time he broke out with small-pox. On account of the ignorance of the family respecting the character of the disease—none of them ever having seen a case of small-pox, or even suspecting any opportunity the boy may have had for taking it—I was not sent for until the disease had made considerable progress.

When I visited him he had been sick several days—the eruption being in the vesicular stage, ready to enter the pustular state. His face was greatly swollen, the eyes nearly closed, and the disease assuming the confluent condition. As soon as I entered the door of the cabin, where the boy was in bed, I announced to those present that it was a case of well developed small-pox. This announcement, of course, produced quite a shock to the family, and great alarm. There were fourteen in the family, including whites and blacks, and none of them had been vaccinated. The mother of the master of the house was nearly seventy years old. I fortunately had some vaccine matter with me, and called them all up and vaccinated them. The mother of the sick boy had a sucking baby, six months old. I now quarantined the cabin of the patient, and ordered no one to enter, save the mother, who was to act as the nurse, and of course had to take care of her child.

Not knowing, as before remarked, that the boy was afflicted with such a terrible disease as small-pox, all the members of the family had been in the cabin every day to see him. Even when they sent for me they did not know the nature of his trouble. In due time, fortunately, all the family, except the nursing child, became under the influence of the vaccine matter. In common parlance, they all *took* finely, and there was not a symptom of variola or varioloid manifested in any of them except the baby.

On account of my great apprehensions of the spreading of the disease, I took the precaution to put the family on a close dietary regimen, from which fact, no doubt, the case of the child proved to be quite mild. The boy and child both got well.

The happy result growing out of the immediate vaccination of this family scarcely needs any comment. It not only prevented the further development of the terrible scourge of small-pox, but also allayed the great consternation which was prevalent in the neighborhood.

Another instance of the immediate good effects of vaccine occurred at my house in 1864. My brother-in-law, Dr. Hall, of Maryland, was on a visit to me, when a neighbor doctor called on him. This neighbor physician had just left a small-pox patient, without having changed his clothes; and just thirteen days after this visit Dr. Hall broke out with variola. Of course the danger in this instance was not near so great as in the previous one, as I recognized the disease as soon as the eruption made its appearance. I immediately vaccinated all who had been in the presence of Dr. Hall, and not one had any symptom of small-pox.

About all the objections urged against vaccination by those who are opposed to its practice, are the alleged cases of constitutional diseases which have been charged to its account. It may be possible that syphilitic and tuberculous diseases may be contracted in this way, but I should judge, from all the evidence that can be obtained from reliable data, the cases resulting in this way are very few.

But, even admitting all that the anti-vaccinationists charge against it to be true, we should rather blame the vaccinator than the vaccination. In the first place, it is the duty of the physician, or party who vaccinates, to select his matter from children whose family history give no evidence of hereditary or constitutional disease; then there can be no possible danger resulting from vaccinating with it. But suppose a scab is taken from a child who is possibly affected with some constitutional disease, it does not follow that the matter introduced into the arm of another child should reproduce the trouble. It is a known fact that if the blood and dermoid tissue that may be contained in the scab be excluded, and the virus proper be only used, that there can be no possible danger resulting from its use. The disease, if any exists, resides in those portions of the scab above alluded to. I think all careful physicians who practice vaccination, will use only the virus proper, contained in the scab, and exclude all other elements.

As respects bovine virus, I do not think the same objections could be urged, as against the humanized matter, as most of it that is used is taken from the pock in a fluid state, which necessarily is pure, even if the animal should be diseased. The only way in which disease could be communicated from bovine matter, would be from the scab, and improperly used. Of course it is the duty of all proprietors of bovine stables to select perfectly healthy animals from which to procure vaccine virus.

I presume all who have had much experience in vaccinating, have had more or less trouble during epidemics of erysipelas. I have been compelled to desist from vaccinating in two epidemics on account of erysipelatous inflammation setting up in the little wound. But it cannot be said that this character of inflammation was due to impure virus, as any surgical operation, however slight, would be affected likewise. I well remember (thirty-seven years ago) that epidemic erysipelas was so great in the Louisville hospital that no surgical operation could be performed with safety. Even vaccination or leech-bites would be followed by erysipelas.

Of course all these things are vehemently urged against vaccination by those who oppose it. But is it reasonable to charge such evil results to the account of vaccination when properly performed with the proper kind of matter? It would be just as rational to say that opium and other powerful drugs should be excluded from the Materia Medica because some have committed suicide with them, or have been poisoned by their injudicious administration. Or, to say that ovariotomy should not be performed because a patient now and then dies from its effects. It is not intended, or proper, for anybody and everybody to practice vaccination, any more than it is to practice medicine or perform surgical operations.

Qualification should be made essential in the performance of anything involving life and health; and if the people permit every ignoramus who may present himself, to either vaccinate them or prescribe and give them medicine, they should bear the consequences without complaining. It should not be expected of one unacquainted with the use of a tool to know how to handle it, no more than it is for a layman to prescribe and administer drugs properly.

When we take into account the great annual mortality that resulted from epidemic small-pox throughout the world previous to the discovery of vaccination, and the small amount from that cause since it has been practiced, the arguments of the anti-vaccinationists make but a poor showing. I think it might be asserted without hesitancy, that, if properly performed, with pure virus, either bovine or humanized, vaccination is not only a sure protection against small-pox, but not at all deleterious to the general health.

Admitting these premises to be correct, and I think they cannot be successfully controverted, does it not seem strange that any one, even Mr. Bergh, *himself*, should endeavor to destroy or abate a means so grand and beneficent in its results, and so simple in its use?

Were I asked the question, who hath conferred, through his genius, the greatest boon upon our race; not only from the ranks of our own profession, (full of noble deeds and grand discoveries) but of the world at large? I would answer unhesitatingly, William Jenner.

As to the durability of vaccination, as a prophylactic against variola, it has not been fully determined by the profession. But it is advised by all to repeat vaccination every few years, and especially when small-pox is prevalent. It has been frequently proved that even imperfect vaccination is to some extent a protection; and the more marks one has the better is he protected. If a person who has been even imperfectly vaccinated takes small-pox, his chances for recovery are 40 to 50 per cent. greater than if he had not been vaccinated.

It may be that some persons are protected much longer than others. From the time I was vaccinated up to the time I attended my last case of small-pox, there had intervened a period of about forty-three years; during which time I had not been re-vaccinated. But I would not advise any one to take the same risk. I agree with a majority of my professional brethren, that everybody should be vaccinated whenever small-pox is in their vicinity.

